# The 7th Blind Test of CSP Methods: Triumphs, Challenges and Insights

**DOI:** 10.1063/4.0001007

**Published:** 2025-10-27

**Authors:** Susan M Reutzel-Edens, Lily M Hunnisett

**Affiliations:** l SuRE Pharma Consulting, LLC, Zionsville, IN, USA; 2 Cambridge Crystallographic Data Centre, Cambridge, CB2 1EZ, United Kingdom

## Abstract

Crystalline forms are of high interest to industry and academia alike for the exquisite control they confer over physicochemical properties affecting functional material performance, the quality, safety and efficacy of medicines, as well as their collective value as intellectual property. Without *a priori* knowledge of how molecular properties may be impacted by crystal structure or a ‘sure-fire’ approach to experimentally finding crystal forms, interest has turned to predicting how molecules can crystallize, starting with the accurate prediction of crystal structure. As one of the most challenging problems we face in materials science, and once considered an impossible task, crystal structure prediction (CSP) has undergone an explosive evolution in recent years, leading the way from ‘simulation’ towards ‘prediction’.

The seventh blind test of Crystal Structure Prediction[1][2], a community initiative coordinated by the Cambridge Crystallographic Data Centre (CCDC), provided an opportunity to benchmark CSP methods against unpublished data and involved both industrial and academic groups from around the globe. This talk highlights the triumphs and challenges highlighted by this initiative and discusses the importance of CSP and its applications within the crystallographic community.
